# Effects of Ginger on clinical manifestations and paraclinical features of patients with Severe Acute Respiratory Syndrome due to COVID-19: A structured summary of a study protocol for a randomized controlled trial

**DOI:** 10.1186/s13063-020-04765-6

**Published:** 2020-10-09

**Authors:** Omid Safa, Mehdi Hassaniazad, Mehdi Farashahinejad, Parivash Davoodian, Habib Dadvand, Soheil Hassanipour, Mohammad Fathalipour

**Affiliations:** 1 Department of Clinical Pharmacy, Faculty of Pharmacy, Hormozgan University of Medical Sciences, Bandar Abbas, Iran; 2 Infectious and Tropical Diseases Research Center, Hormozgan Health Institute, Hormozgan University of Medical Sciences, Bandar Abbas, Iran; 3 Gastrointestinal and Liver Diseases Research Center, Guilan University of Medical Sciences, Rasht, Iran; 4 Department of Pharmacology and Toxicology, Faculty of Pharmacy, Hormozgan University of Medical Sciences, Bandar Abbas, Iran; 5 Endocrinology and Metabolic Research Center, Hormozgan University of Medical Sciences, Bandar Abbas, Iran

**Keywords:** COVID-19, Randomized controlled trial, Protocol, Ginger, GI symptoms, C-reactive protein

## Abstract

**Objectives:**

We investigate the effects of Ginger, compared to the usual therapeutic regimen on clinical manifestations and paraclinical features in patients with confirmed COVID-19 that are moderately ill.

**Trial design:**

This is a single center, randomized, double-blind, placebo-controlled clinical trial with parallel group design.

**Participants:**

*Inclusion criteria*:

1. Patients admitted to Severe Acute Respiratory Syndrome (SARS) Departments at Shahid Mohammadi Hospital, Bandar Abbas, Iran

2. Age ≥18 years (weight ≥35 kg)

3. Hospitalized ≤48 hours

4. Confirmed SARS-CoV-2 diagnosis (Positive polymerase chain reaction (PCR))

5. Moderate pneumonia and lung involvement in imaging

6. Signing informed consent and willingness of study participant to accept randomization to any assigned treatment arm

*Exclusion criteria*:

1. Underlying diseases, including heart disease, chronic hypertension, severe renal failure, severe liver failure, and thyroid disorders

2. Use of warfarin, selective serotonin reuptake inhibitors (SSRIs), monoamine oxidase inhibitors (MAOIs), diuretics, corticosteroids, and antiarrhythmic drugs

3. Severe and critical pneumonia

4. History of known allergy to Ginger

5. Pregnancy and breastfeeding

**Intervention and comparator:**

*Intervention group*: The standard treatment regimen for COVID-19 along with Ginger-based herbal tablets (Vomigone ®, Dineh Pharmaceutical Company, Iran) at a dose of 1000 mg three times a day for a period of seven days.

*Control group*: The standard treatment for COVID-19 based on the Iranian Ministry of Health and Medical Education's protocol, along with Vomigone-like placebo tablets (Dineh Pharmaceutical Company, Iran) at a dose of two tablets three times a day for a period of seven days.

**Main outcomes:**

The primary outcome is recovery rate of clinical symptoms, including fever, dry cough, tiredness, and GI symptoms as well as paraclinical features, including thrombocytopenia, lymphocytopenia, and C-reactive protein within seven days of randomization.

Time to improvement of clinical and paraclinical features along with the incidence of serious adverse events are the secondary outcomes within seven days of randomization.

**Randomization:**

An interactive web-based system will be used to allocate eligible participants, based on the inclusion and exclusion criteria, to one of the two study arms (in a 1:1 ratio) using block randomization.

**Blinding (masking):**

All study participants, research coordinators, clinicians, nurses, and investigators will be blinded to the group assignment.

**Numbers to be randomized (sample size):**

A total of 84 participants will be randomized into two groups of 42 patients.

**Trial Status:**

The protocol is Version 1.0, May 23, 2020. Recruitment began July 21, 2020, and is anticipated to be completed by October 30, 2020.

**Trial registration:**

This clinical trial has been registered in the Iranian Registry of Clinical Trials (IRCT). The registration number is “IRCT20200506047323N1”. Registration date is 23 May 2020.

**Full protocol:**

The full protocol is attached as an additional file, accessible from the Trials website (Additional file 1). In the interest in expediting dissemination of this material, the familiar formatting has been eliminated; this Letter serves as a summary of the key elements of the full protocol.

## Supplementary information

**Additional file 1.** Full Study Protocol.

## Data Availability

The corresponding author has access to the final trial information, and the data will be available on reasonable request (Contact: M.fathalipour@hums.ac.ir).

