# Supplementary material for: Effects of Ginger on clinical manifestations and paraclinical features of patients with Severe Acute Respiratory Syndrome due to COVID-19: A structured summary of a study protocol for a randomized controlled trial
Source: Trials. 2020 Oct 9;21:841. doi: 10.1186/s13063-020-04765-6 (PMC7545374; doi:10.1186/s13063-020-04765-6)
Supplement: Supplementary file 1 — Additional file 1. Full Study Protocol. [file 13063_2020_4765_MOESM1_ESM.docx]

**Protocol**

This trial protocol has been provided by the authors to give readers additional information about their work.

**Effects of Ginger on clinical manifestations and paraclinical features of patients with COVID-19: A structured summary of a study protocol for a randomised controlled trial**

Omid Safa ^1^, Mehdi Hassani-Azad ^2^, Mehdi Farashahinejad ^2^, Parivash Davoodian ^2^, Behnoosh Heidari ^2^, Soheil Hassanipour ^3^, Mohammad Fathalipour ^4,5^

^1^ *Department of Clinical Pharmacy, Faculty of Pharmacy, Hormozgan University of Medical Sciences, Bandar Abbas, Iran.*

^2^ *Infectious and Tropical Diseases Research Center, Hormozgan Health Institute, Hormozgan University of Medical Sciences, Bandar Abbas, Iran.*

^3^ *Gastrointestinal and Liver Diseases Research Center, Guilan University of Medical Sciences, Rasht, Iran.*

^4^ *Department of Pharmacology and Toxicology, Faculty of Pharmacy, Hormozgan University of Medical Sciences, Bandar Abbas, Iran.*

^5^ *Endocrinology and Metabolic Research Center, Hormozgan University of Medical Sciences, Bandar Abbas, Iran.*

*Corresponding author*: Mohammad Fathalipour

Department of Pharmacology and Toxicology, Faculty of Pharmacy, Hormozgan University of Medical Sciences, Bandar Abbas, Iran.

*Telephone*: +98-9133962826

*Fax*: +98-7132307591

*Email* *addresses*: [m.fathalipour@Hums.ac.ir](mailto:m.fathalipour@Hums.ac.ir)

**Abstract**

No effective treatment has yet been proven for the coronavirus disease 2019 (COVID-19). Ginger (*Zingiber officinale*) is a strong demulcent that had been effectively used in the management of a number of diseases. The present study aims to assess the effects of ginger on the clinical manifestations and paraclinical features of patients with covid-19.

A perspective randomized placebo-control clinical trial will be conducted on 84 hospitalized adult patients with covid-19 positive test (polymerase chain reaction test or chest CT-scan). Patients will be randomly assigned in a 1:1 ratio to receive either Ginger (1000 mg three times a day) and standard care (lopinavir–ritonavir 200/50 mg twice a day and hydroxychloroquine 200 mg twice a day) for 7 days, or placebo and standard care alone.

The primary outcomes are defined as recovery rate of clinical symptoms, including fever, high respiratory rate, low oxygen saturation, cough, myalgia, Headache, apnea, weakness, diarrhea, abdominal cramps, nausea, and vomiting. Secondary outcomes include the time from randomization to fever reduction and cough relief as well as the rate of oxygen therapy or noninvasive mechanical ventilation during the trial. Complete blood count and serum biochemical parameters will be checked on the first and 7^th^ days. The adverse events and the diagnostic test will be evaluated at the end of study.

**تأثیر زنجبیل بر تظاهرات بالینی و یافته های آزمایشگاهی در بیماران مبتلا به COVID-19: یک کارآزمایی بالینی کنترل شده با دارونما دوسو کور**

**چکیده**

با وجود انجام تعداد زیادی مطالعه شده در زمینه درما بیماری ناشی از ویروس کورونا (COVID-19)، هنوز هیچ داروی موثر دارویی برای درمان بیماران تایید نشده است. زنجبیل (*Zingiber officinale*) یک تسکین دهنده بسیار موثر است که در درمان تعدادی از بیماری ها مورد استفاده قرار گرفته است. هدف از مطالعه حاضر، بررسی اثر ریزوم زنجبیل بر علائم بالینی و یافته های آزمایشگاهی در بیماران مبتلا به COVID-19 است.

این مطالعه به صورت یک کار آزمایی بالینی کنترل شده با دارونما دو سوکور، روی 84 بیمار بزرگسال با تست مثبت COVID-19 (تست PCR و یا Chest CT-scan) انجام می شود. بيماران به صورت تصادفي به نسبت 1:1 در گروه دریافت کننده ریزوم زنجبیل (1000 ميلي گرم سه بار در روز) و درمان استاندارد (لوپيناویر-ريتونوویر 200/50 میلی گرم دو بار در روز به همراه هیدروکسی کلروکین 200 میلی گرم دو بار در روز) یا گروه دریافت کننده دارونما به همراه درمان استاندارد به مدت 7 روز، قرار خواهند گرفت.

پیامد های اولیه شامل میزان بهبودی علائم بالینی شامل تب، فرکانس تنفس بالا، اشباع کم اکسیژن شریانی، سرفه، درد عضلانی، سردرد، تنگی نفس، ضعف و بی حالی، اسهال، اسپاسم شکمی، تهوع و استفراغ می باشد. پیامد های ثانویه شامل فاصله زمانی شروع مطالعه تا کاهش تب و کاهش سرفه و همچنین میزان نیار به اکسیژن درمانی یا تهویه مکانیکی غیر تهاجمی در طول آزمایش خواهد بود. شمارش کامل سلول های خون و پارامترهای بیوشیمیایی سرم در روزهای اول و هفتم مطالعه بررسی می شود. عوارض جانبی و آزمایش تشخیصی در پایان مطالعه ارزیابی می شود.

**مقدمه**

بیماری کووید ۱۹ (COVID-19^[[1]](#footnote-1)^) که به آن بیماری تنفسی حاد ناشی از کرونا ویروس (SARS-CoV-2^[[2]](#footnote-2)^) نیز گفته می‌شود، بیماری‌ای عفونی است که در دسامبر سال 2019، در ووهان چین پدیدار گردید و به سرعت در چین و خارج از آن شیوع یافت (1, 2). به دنبال شیوع این بیماری در سایر نقاط جهان، سازمان بهداشت جهانی^[[3]](#footnote-3)^ در مارس سال 2020 اپیدمی COVID-19 را به عنوان پاندمی اعلام کرد (3).

شایع ترین علائم این بیماری شامل تب، سرفه و تنگی نفس می باشد(4). با این حال، علائم دیگری همچون خستگی مفرط، درد عضلانی، سردرد، ضعف و بی حالی، کاهش حس بویایی و چشایی، اسهال، اسپاسم شکمی، تهوع و استفراغ ممکن است در بیماران بروز پیدا کند (4-6). علائم به طور معمول 5 روز پس از تماس با ویروس شروع می شوند (7). در حالی که اکثر بیماران دچار علائم خفیف می شوند، در برخی موارد این بیماری به پنمونی ویروسی^[[4]](#footnote-4)^ و نارسایی چند اندامی^[[5]](#footnote-5)^ منجر می شود (8). طبق مطالعاتی که اخیرا در چین انجام شده است، حدود 80 درصد از بیماران مبتلا، بیماری خفیف را تجربه می کنند و میزان مرگ و میر حدود 3/2 درصد است. اما در بیماران 70 تا 79 ساله این میزان به 8/14 درصد و در افراد بالای80 سال به 0/18 درصد افزایش می یابد (9). در 27 آوریل 2020، حدود 3 میلیون مورد ابتلا به این بیماری در 210 کشور گزارش شده است، که مرگ بیش از 206000 نفر را به همراه داشته است.

بر این اساس، تعداد چشم گیری از ناقلان بدون علامت در جمعیت وجود دارد، و بنابراین احتمالاً میزان مرگ و میر بیش از حد ارزیابی می شود. نهایتاٌ، یک درمان مؤثر برای معالجه بیماران علامت دار در جهت کاهش میزان مرگ و میر به ویژه در گروه های پرخطر، تخفیف علایم بیماری و کاهش احتمال انتقال ویروس در جامعه یک نیاز فوری قلمداد می شود. در حال حاضر تعداد زیادی کار آزمایی بالینی جهت بررسی اثرات داروهای قدیمی ضد ویروس، ضد انگل و ضد باکتری در حال انجام است، زیرا اطلاعات فراوانی در زمینه ایمنی، عوارض جانبی، بیواکی والانسی و تداخلات دارویی این دارو ها در دسترس است. علی رغم تلاش های فراوان در این زمینه تا کنون هیچ واکسن و یا رژیم دارویی در درمان این بیماری توسط سازمان های نظارتی دنیا تائید نشده است (10, 11).

در میان داروهای کاندید برای درمان بیماری COVID-19، استفاده از گیاهان دارویی که در طب سنتی هم جهت درمان بیماری های تنفسی استفاده می شوند، یک استراتژی جالب است که مورد توجه محققین قرار گرفته است. زنجبیل^[[6]](#footnote-6)^ (*Zingiber officinale*) گیاهی است که پودر ریزوم آن مصرف درمانی داشته و در طب سنتی اکثر کشور های جهان به فور مورد استفاده قرار می گرفته است (12). آن به عنوان ماده ای با منشاء گیایه دارای اثرات آنتی اکسیدان و ضد التهاب می باشد و در درمان تهوع و استفراغ، نفخ، سوء هاضمه، کولیک و اسهال موثر می باشد. علاوه بر این تجویز آن در درمان بیماری هایی همچون سرماخوردگی، آنفولانزا، رماتیسم و آلزایمر اثرات امید بخشی نشان داده است. مطالعات بالینی در این زمینه نشان داده اند زنجبیل برای تسکین تهوع ناشی از اعمال جراحی، شیمی درمانی و تهوع دوارن بارداری مفید است (13).

مطالعات بالینی متعددی بر عصاره زنجبیل انجام شده است و ایمنی آن در تمامی مطالعات در دوز های مورد مطالعه به اثبات رسیده است. به طور کلی در مصرف این گیاه عارضه جانبی خاصی مشاهده نمی گردد و فقط ممکن است درافراد حساس به زنجبیل، عوارض ملایم گوارشی مشاهده گردد. با این وجود توصیه می گردد در مصرف همزمان مقادیر زیاد زنجبیل با داروهای ضد انعقاد خون مانند وارفارین و در کسانی که دچار اختلالات انعقادی می باشند، احتیاط شود. علاوه بر این توصیه می شود از تجویز زنجبیل در بیماران مبتلا به زخم معده و سنگ صفرا بعلت افزایش ترشح صفرا خودداری شود.

مطالعه حاضر با هدف بررسی اثر بخشی ریزوم گیاه زنجبیل در بیماران مبتلا به بیماری COVID-19 بستری شده در بیمارستان شهید محمدی شهرستان بندرعباس انجام خواهد شد.

**بررسی متون**

زنجبیل با نام علمی*Zingiber officinale*  از خانواده Zingibracea از جمله گیاهان دارویی است که ریزوم آن در طب سنتی کاربرد وسیع دارد و از دیرباز زنجبیل علاوه بر این که به عنوان یک مکمل غذایی استفاده می شده است، در علم پزشکی نیز کاربرد فراوانی داشته است. از دهه هاي قبل به دلیل تنوع ترکیبات فعال آن در درمان بیماري هاي مختلف کاربرد داشته است. اثرات درمانی این گیاه در تعدادی زیادی از بیماری ها از جمله روماتیسم، آسم، تهوع، استفراغ، پرفشاري خون، دمانس، تب و عفونت بررسی شده است. اعمال فارماکولوژیک این گیاه شامل اثرات آنتی اکسیدان، ضدتومور، ضدآپپتوز، ضد التهاب، ضد هیپرگلیسمیا، ضدسرفه و بهبود سرماخوردگی می باشد. ترکیبات فیتوشیمیایی زنجبیل در مطالعات گذشته به طور وسیع شناخته شده است که این ترکیبات شامل؛ روغن هاي اساسی، ترکیبات فنلی، کربوهیدرات ها، پروتئین ها، آلکالوئید ها، گلیکوزید ها، استروئید ها، ترپنوئید ها، ساپونین ها و تانن ها هستند که نقش مهمی در خصوصیات طبی این گیاه ایفا می کنند (14, 15). مواد موثره این گیاه بسته به منطقه ی رویش گیاه بسیار متفاوت است و شامل زینجیبرن، آرکورکومین، بتابیزابولن، نرال، ژالنیال، کامفر، بتافلاندرن، ژرانیال، لینالول و جینجرول ها می باشد.

در بررسی اثرات آنتی اکسیدانی زنجبیل در موش های صحرایی نوزاد مبتلا به انتروکولیت نکروزان^[[7]](#footnote-7)^ مشخص شده است این گیاه می تواند باعث کاهش سطوح TNF-α^[[8]](#footnote-8)^، IL-1β^[[9]](#footnote-9)^ و IL-6^[[10]](#footnote-10)^ شود. بررسی های هیستوپاتولوژیک نیز مشخص کرد که گروه درمان با زنجبیل شدت آسیب های روده ای را کمتر می کند. همچنین تجویز زنجبیل باعث کاهش معنی داری در شاخص های استرس اکسیداتیو حیوانات شده است (16). در یک بررسی که بر روی کراتینوسیت های انسانی انحجام شده است، مشخص شد زنجبیل می تواند سطح رادیکال های آزاد اکسیژن را به دنبال تابش اشعه ماوراء بنفش بر این سلول ها کاهش دهد (17). در بررسی اثرات ضد التهابی زنجبیل بر کولیت القاء شده با دکستران سولفات در موش های صحرایی مشخص شد که این گیاه می تواند میزان فعالیت آنزیم مایلوپراکسیداز و میزان سایتوکاین های التهابی همچون IL-6، IL-1 و TNF-α به صورت معنی داری کاهش دهد (18).

در مطالعه دیگری اثرات ضد التهابی زنجبیل بر روی التهاب بافت کبد القاء شده با دی اتیل نیتروز آمین در موش های صحرایی بررسی شد و مشخص گردید این گیاه می تواند سطح گلوتاتیون را حفظ نماید و باعث بهبود شاخص های التهابی و استرس اکسیداتیو و افزایش سطح Nrf2^[[11]](#footnote-11)^ شود (19). همچنین زنجبیل در مدل حیوانی آسم مشخص شده است که می تواند باعث کاهش بروز علائم این بیماری شود که این اثرات از طریق کاهش تولید سایتوکاین های پیش التهابی و کاهش فعالیت T-cell ها می باشد (20). مطالعه ای که اثر زنجبیل بر بافت ریه حیوانات آزمایشگاهی در معرض طولانی مدت با اتانول را بررسی کرده است نشان می دهد، مصرف عصاره ی این گیاه منجر به کاهش سرعت افزایش آسیب بافتی می شود و اثرات مخرب استرس اکسیداتیو ناشی از اتانول را می کاهد (21). علاوه بر این زنجبیل می تواند موجب کاهش انقباضات نای ناشی از تحریک با کارباکول در موش های صحرایی شوند. بررسی های دقیق تر در این زمینه نشان داد که مکانیسم دقیق این اثر از طریق تحریک احتمالی گیرنده های بتا آدرنرژیک سیستم سمپاتیک می باشد (22). اثرات برنکودیلاسیون زنجبیل در محیط های برون تنی^[[12]](#footnote-12)^ نیز به اثبات رسیده است (23).

مطالعه اثرات زنجبیل بر التهاب بافت ریوی در موش ها صحرایی نشان داده است که این اثرات میتواند از طریق کاهش تجمع ائوزینوفیل ها، سطح IL-4 و IL-5 در مقایسه با گروه کنترل می شود (24). در یک بررسی مشخص شده است ترکیبات 6-شوگائول^[[13]](#footnote-13)^ و 6-جینجرول^[[14]](#footnote-14)^ به عنوان فلاونوئید های زنجبیل موجب اثرات ضد تب و ضد درد می شود و در کنار این اثرات، 6-شوگائول در مقایسه با دی هیدروکدئین فسفات به صورت چشمگیری باعث بهبود سرفه شده است (25).

از سوی دیگر مطالعات نشان داده اند اسانس زنجبیل خاصیت ضد ویروس هرپس سیمپلکس^[[15]](#footnote-15)^ را دارد. ترکیبات موجود در اسانس این گیاه می تواند باعث غیر فعال شدن پروتئین ویروسی CpHV-1^[[16]](#footnote-16)^ می شود. همچنین موجب تخریب پوشش ویروسی^[[17]](#footnote-17)^ شده و ساختارهای لازم برای ورود ویروس به سلول میزبان را نیز مختل می کند (26). علاوه بر این اثرات ضد ویروس سین سیشال تنفسی^[[18]](#footnote-18)^ این گیاه در مدل های برون تنی دیگر نیز نشان داد ه شده است (27).

درکارآزمایی های بالینی انجام شده بر روی ریزوم زنجبیل اثرات مفیدی همچون اثرات ضد دردی، ضد التهاب، ضد تهوع و استفراغ، تنظیم قند خون و فشار خون به اثبات رسیده است.

اثرات ضد التهاب و آنتی اکسیدان این گیاه در مطالعه ای بر روی افراد مبتلا به بیماری سل بررسی شده است. نتایج این مطالعه نشان داده است مصرف پودر ریزوم این گیاه به میزان 3000 میلی گرم در روز به مدت 30 روز در کنار رژیم استاندارد درمان سل باعث بهبود پارامتر های التهابی و اکسیدانی سرم این افراد شده است (28). علاوه بر این مصرف زنجبیل به میزان 1000 میلی گرم در روز به مدت 4 هفته در افراد مبتلا به سل می تواند به طور معنی داری از عوارض گوارشی و سمیت کبدی ناشی از داروهای ضد سل پیش گیری کند (29).

در مطالعه انجام شده بر روی 168 دانش آموز دختر، مشخص شده است که مصرف 200 میلی گرم از پودر ریزوم این گیاه برای 4 بار در روز می تواند به اندازه نوافن در کاهش علائم دیس منوره^[[19]](#footnote-19)^ موثر باشد (30). علی رغم این که در مطالعات متعدد دیگری نیز مشخص شده است که مصرف این گیاه به اندازه دارو های ضد التهاب غیر استروئیدی^[[20]](#footnote-20)^ در کاهش علائم دیس منوره موثر می باشند (30-32)، نتایج حاصل از یک کارآزمایی بالینی تشان می دهد مصرف این گیاه به اندازه تمرین های کششی^[[21]](#footnote-21)^ نمی تواند در بهبود علائم دیس منوره موثر باشد (33).

مصرف زنجبیل به میزان 1500 میلی گرم برای 12 هفته در بیماران مبتلا به روماتیسم مفصلی^[[22]](#footnote-22)^ اثرات ضد التهاب خوبی را به همراه داشته است که این اثرات ناشی از افزایش بیان ژن FoxP3^[[23]](#footnote-23)^ و کاهش بیان ژن های RORγt^[[24]](#footnote-24)^ و T-bet^[[25]](#footnote-25)^ بوده است (34). در مطالعه ای دیگر در این زمینه مشخص شده است که مصرف این گیاه به میزان 500 میلی گرم در روز برای 3 ماه می تواند باعث کاهش پارامتر های التهابی همچون IL-1βو TNF-α در بیماران مبتلا به استئوآرتریت^[[26]](#footnote-26)^ شود (35). علاوه بر این، تعدادی کار آزمایی بالینی اثرات ضد درد این گیاه را در بیماران مبتلا به سر درد های میگرنی بررسی کرده اند. در حالی که اثرات امید بخش این گیاه در برخی مطالعات در پیش گیری از بروز علائم میگرن همانند درمان استاندارد (سوماتریپتان) بوده است (36)، در مطالعات دیگر مصرف روزانه 600 میلی گرم زنجبیل نتوانسته در مقایسه با گروه کنترل (دارونما) اثرات مفیدی بیشتری اعمال کند (36).

در مطالعه ای دیگر که بر روی زنان باردار مبتلا به بیماری تهوع بارداری^[[27]](#footnote-27)^ انجام شده است، مشخص شده مصرف 1000 میلی گرم زنجبیل برای 4 روز اثرات ضد تهوع و استفراغ چشمگیری در مقایسه با گروه کنترل دارد، در حالی که عوارض جانبی خاصی از این گیاه دیده نشده است (37). علاوه بر این نتایج مطالعات متعددی نشان می دهد، اثرات ضد تهوع و استفراغ این گیاه در افرادی که تحت شیمی درمانی بوده اند در مقایسه با گروه کنترل بسیار بیشتر می باشد (38-42). همچنین این اثرات در تهوع و استفراغ های بیمارانی که تحت اعمال جراحی قرار گرفته اند، در مقایسه با گروه کنترل بیشتر بوده است (43).

اثرات مفید زنجبیل در تعدادی از بیماری های متابولیک نیز بررسی شده است. مصرف این گیاه به میزان 3000 میلی گرم در روز به مدت 3 ماه در افراد مبتلا به دیابت نوع 2 باعث بهبود شاخص های گلیسمی^[[28]](#footnote-28)^، ظرفیت آنتی اکسیدان، فاکتور های التهابی همچون CRP^[[29]](#footnote-29)^ در مقایسه با گروه کنترل می شود (44). همچنین، مصرف این گیاه به میزان 3000 میلی گرم در روز به مدت 8 هفته در افراد مبتلا به دیابت نوع 2، باعث بهبود اختلال عملکرد اندتلیوم^[[30]](#footnote-30)^ و فشار خون شده است (45). در کارآزمایی دیگری مشخص شده است وقتی این گیاه به میزان 1000 میلی گرم در روز به مدت 10 هفته به بیماران تحت دیالیز صفاقی تجویز می شود، می تواند به صورت معنی داری در مقایسه با گروه کنترل باعث کاهش تری گلیسیرید سرم شود (46).

مطالعه ای که در دوران شیردهی در زمینه زنجبیل انجام شده است، نشان می دهد که مصرف این گیاه به میزان 1000 میلی گرم در روز برای 7 رز پس از زایمان در مقایسه با گروه کنترل به میزان معنی داری باعث افزایش حجم شیر شده است (47).

مطالعات متعدد دیگری نیز اثرات مفید این گیاه در استفاده به صورت فراورده های غیر خوراکی برای درمان استئوآرتریت (ژل موضعی) (48, 49) تهوع استفراغ ناشی از شیمی درمانی (استنشاقی) (50)، تهوع و استفراغ پس از جراحی (استنشاقی) (51)، نشان داده اند.

**اهداف كلي طرح:**

ارزیابی اثرات زنجبیل بر تظاهرات بالینی و یافته های آزمایشگاهی در بیماران مبتلا به COVID-19

**اهداف ويژه‌ي طرح :**

ارزیابی اثرات زنجبیل بر تظاهرات بالینی در بیماران مبتلا به COVID-19

مقایسه اثر بخشی زنجبیل بر تظاهرات بالینی با دارونما در بیماران مبتلا به COVID-19

ارزیابی اثرات زنجبیل بر یافته های آزمایشگاهی در بیماران مبتلا به COVID-19

مقایسه اثر بخشی زنجبیل بر یافته های آزمایشگاهی با دارونما در بیماران مبتلا به COVID-19

**اهداف كاربردي طرح :**

با توجه به این که اثرات مفیدی نظیر اثرات آنتی اکسیدان، ، ضد التهاب، ضد تب، ضد هیپرگلیسمیا، ضدسرفه و بهبود سرماخوردگی از زنجبیل در مطالعات متعدد نشان داده شده است، می توان گفت در درمان کمکی با این گیاه، امکان بهبود کارایی رژیم های درمانی موجود وجود دارد و در صورت حصول پاسخ دهی بهتر نسبت به درمان استاندارد می توان در آینده این گیاه جهت بهبود سریع تر علائم بالینی و یافته های آزمایشگاهی بیماران، به درمان استاندارد اضافه شود.

**فرضيات يا سوالات پژوهش (باتوجه به اهداف طرح) :**

علائم بالینی بیماران مبتلا به COVID-19 در گروه مصرف کننده زنجبیل نسبت به گروه دریافت کننده دارونما بهتر و سریع تر بهبود می یابد.

یافته های آزمایشگاهی بیماران مبتلا به COVID-19 در گروه مصرف کننده زنجبیل نسبت به گروه دریافت کننده دارونما بهتر و سریع تر بهبود می یابد.

**روش اجرای طرح**

**طراحی مطالعه و شرکت کنندگان**

این مطالعه در قالب یک کارآزمایی بالینی تصادفی با برچسب باز^[[31]](#footnote-31)^ بر روی 84 بیمار مبتلا به بیماری COVID-19 بستری شده در بخش سندرم حاد تنفسی بیمارستان شهید محمدی شهرستان بندرعباس، ایران طراحی شده است. بيماران از 25 می 2020 به صورت آينده نگر وارد مطالعه و پيگيري خواهند شد. با توجه به نسبت 1:1 بين گروه آزمايش (پرتکل استاندارد کشوری به همراه فراورده حاوی زنجبیل) و گروه کنترل (پرتکل استاندارد کشوری به همراه دارونما)، بیماران به صورت تصادفی وارد هر یک از بازو های مداخله می شوند.

در شروع مطالعه بیماران بر اساس علائم بالینی و یافته های پاراکلینیکی در گروه های خفیف، متوسط، شدید و بحرانی تقسیم بندی می شوند (جدول 1). معیارهای ورود بیماران شامل؛ (1) سن 18 سال یا بالاتر، (2) رضایت آگاهانه و داوطلبانه، (3) علائم بالینی اولیه و (4) تشخیص قطعی بیماری COVID-19 از طریق تست PCR^[[32]](#footnote-32)^ (و یاChest CT-scan ^[[33]](#footnote-33)^) به همرا شدت متوسط​​، شدید و یا بحرانی، خواهند بود. تمامی بیماران با سابقه ای از (1) هپاتیت مزمن، سیروز کبدی، بیماری های کلستاتیک کبد، التهاب کیسه صفرا و زخم های پپتیک (2) حساسیت به زنجبیل (3) مصرف دارو های ضد انعقاد همچون وارفارین و دارو های هرمونی و (4) زنان در دوران بارداری و شیردهی از مطالعه خارج می شوند.

در ابتدای کارآزمایی، مشخصات عمومی، مشخصات دموگرافیک و سوابق پزشکی بیماران با استفاده از پرسشنامه جمع آوری می شود. پس از ارائه توضیحات کافی و کسب رضایت آگاهانه کتبی از جانب بیمار یا بستگان درجه یک (در بیماران با سطح هوشیاری پایین یا زوال عقل)، بیماران با استفاده از روش تصادفی سازی بلوک ها دو گروه مساوی (گروه مداخله و گروه کنترل) تقسیم می شوند.

**گروه های مداخله**

گروه A بیمارانی هستند که درمان استاندارد بر اساس پروتکل تعیین شده از سوی وزارت بهداشت برای بیماری COVID-19 به همراه دارونما را دریافت می کنند. درمان استاندارد شامل دارو های هیدروکسی کلروکین (200 میلی گرم دو بار در روز) و آزیترومایسین (250 میلی گرم دو بار در روز) به مدت 7 روز می باشد. گروه B بیمارانی هستند که علاوه بر درمان استاندارد فوق الذکر، یک فراورده گیاهی مبتنی بر پودر ریزوم زنجبیل در شکل دارویی قرص، با دوز 1000 میلی گرم سه بار در روز به مدت 7 روز دریافت می کنند. در این مطالعه از قرص های ومیگان (Vomigone) شرکت داروسازی دینه استفاده می شود که هر قرص حاوی 500 میلی گرم میلی گرم پودر ریزوم زنجبیل می باشد. قرص های دارونما نیز توسط شرکت داروسازی دینه با کیفیت قرص های ومیگان تولید شده و با دوز دو قرص سه بار در روز تجویز می شود.

**بررسی پیامد ها**

پیامد های اولیه این مطالعه میزان بهبودی علائم بالینی در طی دوره مداخله نظر گرفته می شود. بهبود علائم بالینی به عنوان بهبود مداوم (بیشتر از 72 ساعت) درجه حرارت بدن، فرکانس تنفس و میزان اشباع اكسيژن خون پس از شروع درمان می باشد که با معيارهاي كمي که در ادامه آمده است تعریف می شود: دمای دهانی ≥ 6/36 درجه سانتیگراد؛ فرکانس تنفسی ≥24 بار در دقیقه و اشباع اکسیژن ≤98 درصد بدون تنفس مکانیکی. علاوه بر این، نیاز به اکسیژن درمانی و تهویه با فشار مثبت غیر تهاجمی در فالوآپ های روزانه به همراه علائم بالینی دیگر علائم بالینی دیگر همچون سرفه، درد عضلانی، سردرد، تنگی نفس، ضعف و بی حالی، کاهش حس بویایی و چشایی، اسهال، اسپاسم شکمی، تهوع و استفراغ به صورت کیفی ثبت می شوند. اندازه گیری مکرر حداقل برای دو بار در هر فالوآپ انجام می شود.

پیامد های ثانویه شامل مدت زمان بستری بودن در بیمارستان، فاصله زمانی از شروع مطالعه (تصادفی سازی در هر یک از گروه های مطالعه) تا کاهش تب (بیماران مبتلا به تب)، فاصله زمانی از شروع مطالعه تا تسکین سرفه (بیماران مبتلا به سرفه متوسط ​​یا شدید در زمان ثبت نام)، فاصله زمانی از شروع مطالعه تا بهبود تنگی نفس، میزان نیاز اکسیژن درمانی کمکی یا تهویه مکانیکی غیر تهاجمی در طول مطالعه، نیاز به بستری شدن در بخش مراقبت های ویژه و میزان نارسایی تنفسی در طول مطالعه (اشباع اکسیژن ≥90 درصد بدون تنفس مکانیکی و یا PaO_2_/FiO_2_ >300 میلی متر جیوه، نیاز به اکسیژن درمانی و یا حمایت تنفسی) می باشند.

انجام آزمایش های CBC^[[34]](#footnote-34)^، شمارش تفریقی سلول های سفید، میزان فریتین، CRP^[[35]](#footnote-35)^، LDH^[[36]](#footnote-36)^، ESR^[[37]](#footnote-37)^، کراتینین و نیتروژن اوره سرم در ابتدای مطالعه و انتهای مطالعه (روز 7 مطالعه و یا زمان ترخیص) بررسی خواهد شد. علاوه بر این تست PCR (و یا Chest CT-scan) و انتهای مطالعه (روز 7 مطالعه و یا زمان ترخیص) بررسی خواهد شد. همچنین واکنش های نا خواسته (به ویژه واکنش های ازدیاد حسیاسیت مربوط به سیستم گوارش)، فراوانی عوارض جانبی احتمالی ناشی از مداخله و فراوانی انصراف از مطالعه به علت عوارض جانبی در گروه های مورد مطالعه به صورت روزانه ثبت می شوند.

**روش محاسبه حجم نمونه**

با استفاده از مطالعات مشابه انجام شده در گروه های چند دارویی میزان بهبودی بالینی مورد انتظار در روز 7 از گروه مورد مطالعه 80 درصد، بهبودی بالینی گروه دریافت کننده دارونما 40 درصد، α = 0.05 ، β = 0.10، قدرت = 90/0 در نظر گرفته شد. با توجه به توزیع 1:1 بین گروه مورد مطالعه و گروه دریافت کننده دارونما، حجم نمونه آماری 34 شرکت کننده در هر گروه است. اندازه نمونه با توجه به عواملی مانند ریختن حدود 20 درصد افزایش یافته است. این کارآزمایی شامل 84 نفر (42 نفر در هر گروه) می باشد.


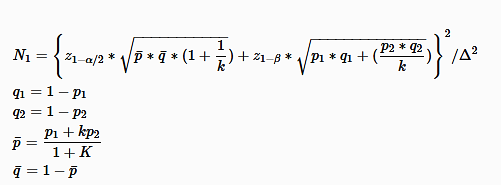

**روش تصادفی سازی بلوک**

در این روش تعداد افراد در هر یک از گروه های مطالعه در طول درمان با یکدیگر برابر است. با توجه به تعداد 84 نفر افراد شرکت کننده (42 نفر در هر گروه) و مدت زمان تقریبی 10 هفته برای تکمیل ورود افراد به مطالعه، از 20 بلوک 4 تایی (در صورت وجود بیمار به تعداد کافی از 8 بلوک 10 تایی) استفاده خواهد شد (جدول 3). روش کار در این نوع از تصادفی سازی شبیه به روش تصادفی سازی ساده است، تنها تعداد افراد در طول دوره درمان در دو گروه مداخله و گروه دریافت کننده دارونما یکسان می باشد. قبل از شروع مداخله، داروها و دارونماها توسط مجری طرح کد گذاری می شوند و بر اساس کدها دارو به بیماران تجویز می شود. در طی مطالعه پزشک معالج، کادر درمانی و بیماران هیچ اطلاعی از نوع مداخله (دارو یا دارونما) نخواهند داشت.

**آنالیز آماری**

برای تحليل آماری از نرم افزار SPSS ورژن 0/18 استفاده می شود. برای مقایسه شاخص های اصلی اثر بخشی (میزان بهبود علائم بالینی) به عنوان پیامد اولیه و پیامد های ثانویه بین گروه مورد مطالعه و گروه دریافت کننده دارونما، از آزمون t برای متغیرهای پیوسته و یا آزمون Wilcoxon (در صورت عدم استفاده از آزمون t) برای متغییر های گسسته رتبه ای استفاده می شود. توصیف آماری متغیرهای کیفی به صورت فراوانی یا درصد مشاهده خواهد بود و برای مقایسه بین گروه ها از آزمون های Chi-square یا Fisher’s exact استفاده خواهد شد. برای کلیه آزمون های آماری، P <0.05 (دو طرفه) از نظر آماری معنی دار در نظر گرفته می شود.

**ملاحظات اخلاقي**

جهت شرکت در این مطالعه از تمامی بیماران رضایت آگاهانه اخذ خواهد شد. اطلاعات مربوطه بصورت محرمانه حفظ خواهد شد. هیچ یک از مشخصات فردی افراد شرکت کننده از جمله اسم و فامیل آنها وارد کامپیوتر نخواهد شد و به تمامی افراد کد پروژه داده شده و آنالیز بر اساس آن انجام خواهد شد. اطلاعات اولیه در فایل های قفل دار و نزد مجری اصلی تا اتمام پروژه و انتشار مقالات باقی خواهد ماند. در هر مرحله از طرح بیماران می توانند بنا به تمایل شخصی از طرح خارج شوند.

**محدوديت‌هاي اجرايي طرح و روش رفع آن ها**

عدم همکاری بیماران که امید است با توضیحات کافی برای آن ها این مشکل رفع شود و قبل از شروع به بیماران در مورد عوارض جانبی بیماری و دارو توضیح داده می شود. از آنها قبل از شروع درمان رضایت نامه کتبی دریافت می شود. همچنین این پژوهش از نظر اخلاق پزشکی مورد تایید است.

**References**

1. Lai C-C, Shih T-P, Ko W-C, Tang H-J, Hsueh P-R. Severe acute respiratory syndrome coronavirus 2 (SARS-CoV-2) and corona virus disease-2019 (COVID-19): the epidemic and the challenges. International journal of antimicrobial agents. 2020:105924.

2. Wang L-s, Wang Y-r, Ye D-w, Liu Q-q. A review of the 2019 Novel Coronavirus (COVID-19) based on current evidence. International Journal of Antimicrobial Agents. 2020:105948.

3. Organization WH. WHO Director-General's opening remarks at the media briefing on COVID-19-11 March 2020. Geneva, Switzerland. 2020.

4. Kailas Khandu Sanap D, Sanap AK. What We Know So Far About New Coronavirus (COVID-19). Sustainable Humanosphere. 2020;16(1):1470-6.

5. Magdi H. COVID-19 Coronavirus Disease. 2020.

6. Hopkins C, Kumar N. Loss of sense of smell as marker of COVID-19 infection. ENT UK at The Royal College of Surgeons of England. 2020.

7. Velavan TP, Meyer CG. The COVID-19 epidemic. Trop Med Int Health. 2020;25(3):278-80.

8. Hui DS, Azhar EI, Madani TA, Ntoumi F, Kock R, Dar O, et al. The continuing 2019-nCoV epidemic threat of novel coronaviruses to global health—The latest 2019 novel coronavirus outbreak in Wuhan, China. International Journal of Infectious Diseases. 2020;91:264.

9. Wu Z, McGoogan JM. Characteristics of and important lessons from the coronavirus disease 2019 (COVID-19) outbreak in China: summary of a report of 72 314 cases from the Chinese Center for Disease Control and Prevention. Jama. 2020.

10. Colson P, Rolain J-M, Lagier J-C, Brouqui P, Raoult D. Chloroquine and hydroxychloroquine as available weapons to fight COVID-19. Int J Antimicrob Agents. 2020;105932(10.1016).

11. Colson P, Rolain J-M, Raoult D. Chloroquine for the 2019 novel coronavirus. Int J Antimicrob Agents. 2020.

12. Langner E, Greifenberg S, Gruenwald J. Ginger: history and use. Advances in therapy. 1998;15(1):25-44.

13. Abo-Esa JF. Study on some ectoparasitic diseases of catfish, Clarias gariepinus with their control by ginger, Zingiber officiale. Mediterranean Aquaculture Journal. 2008;1(1):1-9.

14. Prakash J. Chemical composition and antioxidant properties of ginger root (Zingiber officinale). Journal of Medicinal Plants Research. 2010;4(24):2674-9.

15. Otunola GA, Oloyede OB, Oladiji AT, Afolayan AJ. Comparative analysis of the chemical composition of three spices–Allium sativum L. Zingiber officinale Rosc. and Capsicum frutescens L. commonly consumed in Nigeria. African Journal of Biotechnology. 2010;9(41):6927-31.

16. Cakir U, Tayman C, Serkant U, Yakut HI, Cakir E, Ates U, et al. Ginger (Zingiber officinale Roscoe) for the treatment and prevention of necrotizing enterocolitis. J Ethnopharmacol. 2018;225:297-308.

17. Chen F, Tang Y, Sun Y, Veeraraghavan VP, Mohan SK, Cui C. 6-shogaol, a active constiuents of ginger prevents UVB radiation mediated inflammation and oxidative stress through modulating NrF2 signaling in human epidermal keratinocytes (HaCaT cells). Journal of photochemistry and photobiology B, Biology. 2019;197:111518.

18. Kim MS, Kim JY. Ginger attenuates inflammation in a mouse model of dextran sulfate sodium-induced colitis. Food science and biotechnology. 2018;27(5):1493-501.

19. Mansour DF, Abdallah HMI, Ibrahim BMM, Hegazy RR, Esmail RSE, Abdel-Salam LO. The Carcinogenic Agent Diethylnitrosamine Induces Early Oxidative Stress, Inflammation and Proliferation in Rat Liver, Stomach and Colon: Protective Effect of Ginger Extract. Asian Pacific journal of cancer prevention : APJCP. 2019;20(8):2551-61.

20. Yocum GT, Hwang JJ, Mikami M, Danielsson J, Kuforiji AS, Emala CW. Ginger and its bioactive component 6-shogaol mitigate lung inflammation in a murine asthma model. American journal of physiology Lung cellular and molecular physiology. 2020;318(2):L296-l303.

21. Shirpoor A, Gharalari FH, Rasmi Y, Heshmati E. Ginger extract attenuates ethanol-induced pulmonary histological changes and oxidative stress in rats. Journal of biomedical research. 2017.

22. Mangprayool T, Kupittayanant S, Chudapongse N. Participation of citral in the bronchodilatory effect of ginger oil and possible mechanism of action. Fitoterapia. 2013;89:68-73.

23. Townsend EA, Siviski ME, Zhang Y, Xu C, Hoonjan B, Emala CW. Effects of ginger and its constituents on airway smooth muscle relaxation and calcium regulation. American journal of respiratory cell and molecular biology. 2013;48(2):157-63.

24. Ahui ML, Champy P, Ramadan A, Pham Van L, Araujo L, Brou Andre K, et al. Ginger prevents Th2-mediated immune responses in a mouse model of airway inflammation. International immunopharmacology. 2008;8(12):1626-32.

25. Suekawa M, Ishige A, Yuasa K, Sudo K, Aburada M, Hosoya E. Pharmacological studies on ginger. I. Pharmacological actions of pungent constitutents, (6)-gingerol and (6)-shogaol. Journal of pharmacobio-dynamics. 1984;7(11):836-48.

26. Camero M, Lanave G, Catella C, Capozza P, Gentile A, Fracchiolla G, et al. Virucidal activity of ginger essential oil against caprine alphaherpesvirus-1. Veterinary microbiology. 2019;230:150-5.

27. Chang JS, Wang KC, Yeh CF, Shieh DE, Chiang LC. Fresh ginger (Zingiber officinale) has anti-viral activity against human respiratory syncytial virus in human respiratory tract cell lines. Journal of Ethnopharmacology. 2013;145(1):146-51.

28. Kulkarni RA, Deshpande AR. Anti-inflammatory and antioxidant effect of ginger in tuberculosis. Journal of complementary & integrative medicine. 2016;13(2):201-6.

29. Emrani Z, Shojaei E, Khalili H. Ginger for Prevention of Antituberculosis-induced Gastrointestinal Adverse Reactions Including Hepatotoxicity: A Randomized Pilot Clinical Trial. Phytotherapy research : PTR. 2016;30(6):1003-9.

30. Adib Rad H, Basirat Z, Bakouei F, Moghadamnia AA, Khafri S, Farhadi Kotenaei Z, et al. Effect of Ginger and Novafen on menstrual pain: A cross-over trial. Taiwanese journal of obstetrics & gynecology. 2018;57(6):806-9.

31. Jenabi E. The effect of ginger for relieving of primary dysmenorrhoea. JPMA The Journal of the Pakistan Medical Association. 2013;63(1):8-10.

32. Shirvani MA, Motahari-Tabari N, Alipour A. The effect of mefenamic acid and ginger on pain relief in primary dysmenorrhea: a randomized clinical trial. Archives of gynecology and obstetrics. 2015;291(6):1277-81.

33. Shirvani MA, Motahari-Tabari N, Alipour A. Use of ginger versus stretching exercises for the treatment of primary dysmenorrhea: a randomized controlled trial. Journal of integrative medicine. 2017;15(4):295-301.

34. Aryaeian N, Shahram F, Mahmoudi M, Tavakoli H, Yousefi B, Arablou T, et al. The effect of ginger supplementation on some immunity and inflammation intermediate genes expression in patients with active Rheumatoid Arthritis. Gene. 2019;698:179-85.

35. Mozaffari-Khosravi H, Naderi Z, Dehghan A, Nadjarzadeh A, Fallah Huseini H. Effect of Ginger Supplementation on Proinflammatory Cytokines in Older Patients with Osteoarthritis: Outcomes of a Randomized Controlled Clinical Trial. Journal of nutrition in gerontology and geriatrics. 2016;35(3):209-18.

36. Maghbooli M, Golipour F, Moghimi Esfandabadi A, Yousefi M. Comparison between the efficacy of ginger and sumatriptan in the ablative treatment of the common migraine. Phytotherapy research : PTR. 2014;28(3):412-5.

37. Sharifzadeh F, Kashanian M, Koohpayehzadeh J, Rezaian F, Sheikhansari N, Eshraghi N. A comparison between the effects of ginger, pyridoxine (vitamin B6) and placebo for the treatment of the first trimester nausea and vomiting of pregnancy (NVP). The journal of maternal-fetal & neonatal medicine : the official journal of the European Association of Perinatal Medicine, the Federation of Asia and Oceania Perinatal Societies, the International Society of Perinatal Obstet. 2018;31(19):2509-14.

38. Ansari M, Porouhan P, Mohammadianpanah M, Omidvari S, Mosalaei A, Ahmadloo N, et al. Efficacy of Ginger in Control of Chemotherapy Induced Nausea and Vomiting in Breast Cancer Patients Receiving Doxorubicin-Based Chemotherapy. Asian Pacific journal of cancer prevention : APJCP. 2016;17(8):3877-80.

39. Arslan M, Ozdemir L. Oral intake of ginger for chemotherapy-induced nausea and vomiting among women with breast cancer. Clinical journal of oncology nursing. 2015;19(5):E92-7.

40. Sanaati F, Najafi S, Kashaninia Z, Sadeghi M. Effect of Ginger and Chamomile on Nausea and Vomiting Caused by Chemotherapy in Iranian Women with Breast Cancer. Asian Pacific journal of cancer prevention : APJCP. 2016;17(8):4125-9.

41. Li X, Qin Y, Liu W, Zhou XY, Li YN, Wang LY. Efficacy of Ginger in Ameliorating Acute and Delayed Chemotherapy-Induced Nausea and Vomiting Among Patients With Lung Cancer Receiving Cisplatin-Based Regimens: A Randomized Controlled Trial. Integrative cancer therapies. 2018;17(3):747-54.

42. Marx W, McCarthy AL, Ried K, McKavanagh D, Vitetta L, Sali A, et al. The Effect of a Standardized Ginger Extract on Chemotherapy-Induced Nausea-Related Quality of Life in Patients Undergoing Moderately or Highly Emetogenic Chemotherapy: A Double Blind, Randomized, Placebo Controlled Trial. Nutrients. 2017;9(8).

43. Kamali A, Beigi S, Shokrpour M, Pazuki S. The Efficacy Of Ginger And Doxedetomidine In Reducing Postoperative Nausea And Vomiting In Patients Undergoing Abdominal Hysterectomy. Alternative therapies in health and medicine. 2020;26(2):28-33.

44. Shidfar F, Rajab A, Rahideh T, Khandouzi N, Hosseini S, Shidfar S. The effect of ginger (Zingiber officinale) on glycemic markers in patients with type 2 diabetes. Journal of complementary & integrative medicine. 2015;12(2):165-70.

45. Azimi P, Ghiasvand R, Feizi A, Hosseinzadeh J, Bahreynian M, Hariri M, et al. Effect of cinnamon, cardamom, saffron and ginger consumption on blood pressure and a marker of endothelial function in patients with type 2 diabetes mellitus: A randomized controlled clinical trial. Blood pressure. 2016;25(3):133-40.

46. Tabibi H, Imani H, Atabak S, Najafi I, Hedayati M, Rahmani L. Effects of Ginger on Serum Lipids and Lipoproteins in Peritoneal Dialysis Patients: A Randomized Controlled Trial. Peritoneal dialysis international : journal of the International Society for Peritoneal Dialysis. 2016;36(2):140-5.

47. Paritakul P, Ruangrongmorakot K, Laosooksathit W, Suksamarnwong M, Puapornpong P. The Effect of Ginger on Breast Milk Volume in the Early Postpartum Period: A Randomized, Double-Blind Controlled Trial. Breastfeeding medicine : the official journal of the Academy of Breastfeeding Medicine. 2016;11:361-5.

48. Tosun B, Unal N, Yigit D, Can N, Aslan O, Tunay S. Effects of Self-Knee Massage With Ginger Oil in Patients With Osteoarthritis: An Experimental Study. Research and theory for nursing practice. 2017;31(4):379-92.

49. Amorndoljai P, Taneepanichskul S, Niempoog S, Nimmannit U. A Comparative of Ginger Extract in Nanostructure Lipid Carrier (NLC) and 1% Diclofenac Gel for Treatment of Knee Osteoarthritis (OA). Journal of the Medical Association of Thailand = Chotmaihet thangphaet. 2017;100(4):447-56.

50. Lua PL, Salihah N, Mazlan N. Effects of inhaled ginger aromatherapy on chemotherapy-induced nausea and vomiting and health-related quality of life in women with breast cancer. Complementary therapies in medicine. 2015;23(3):396-404.

51. Adib-Hajbaghery M, Hosseini FS. Investigating the effects of inhaling ginger essence on post-nephrectomy nausea and vomiting. Complementary therapies in medicine. 2015;23(6):827-31.

**جدول 1. تقسیم بندی بیماران مبتلا به COVID-19**

|  | **Disease category** | **Definition(s)** |
| --- | --- | --- |
| 1 | Mild | The clinical symptoms are mild and no pneumonia on imaging |
| 2 | Moderate | With fever, respiratory tract and other symptoms, imaging shows pneumonia |
| 3 | Severe | Meet any of the following:  (a) Shortness of breath, RR> 30 times/minute  (b) Finger oxygen saturation is < 93% at rest  (c) Arterial blood oxygen partial pressure (PaO2)/oxygen concentration (FiO2) < 300mmHg (l mmHg = 0.133 kPa)  At high altitudes (above 1000 meters), PaO2/FiO2 should be corrected according to the following formula: PaO2/FiO2 X [Atmospheric pressure (mmHg)/760] |
| 4 | Critical | One of the following:  (a) Respiratory failure and requires mechanical ventilation  (b) Shock  (c) Combining other organ failures requires ICU monitoring and treatment |

**جدول 2. تصادفی سازی بلوک ها**

| B | A | B | A | A | B | B | A | Block 1 |
| --- | --- | --- | --- | --- | --- | --- | --- | --- |
|  |  |  |  |  |  |  |  | Block 2 |
|  |  |  |  |  |  |  |  | Block 3 |
|  |  |  |  |  |  |  |  | Block 4 |
|  |  |  |  |  |  |  |  | Block 5 |
|  |  |  |  |  |  |  |  | Block 6 |
|  |  |  |  |  |  |  |  | Block 7 |
|  |  |  |  |  |  |  |  | Block 8 |
|  |  |  |  |  |  |  |  | Block 9 |
|  |  |  |  |  |  |  |  | Block 10 |

A: گروه مداخله و B: گروه دریافت کننده دارونما

1. Coronavirus disease 2019 [↑](#footnote-ref-1)
2. Severe acute respiratory syndrome coronavirus 2 [↑](#footnote-ref-2)
3. World Health Organization [↑](#footnote-ref-3)
4. Viral pneumonia [↑](#footnote-ref-4)
5. Multiple organ failure [↑](#footnote-ref-5)
6. Ginger [↑](#footnote-ref-6)
7. Necrotizing enterocolitis [↑](#footnote-ref-7)
8. Tumor necrosis factor alpha [↑](#footnote-ref-8)
9. Interleukin 1 beta [↑](#footnote-ref-9)
10. Interleukin 6 [↑](#footnote-ref-10)
11. Nuclear factorerythroid 2-related factor2 [↑](#footnote-ref-11)
12. *in vitro* [↑](#footnote-ref-12)
13. 6-Shogaol [↑](#footnote-ref-13)
14. 6-Gingerol [↑](#footnote-ref-14)
15. Herpes simplex virus [↑](#footnote-ref-15)
16. Caprine alphaherpesvirus [↑](#footnote-ref-16)
17. Viral envelope [↑](#footnote-ref-17)
18. Respiratory syncytial virus [↑](#footnote-ref-18)
19. Dysmenorrhea [↑](#footnote-ref-19)
20. Nonsteroidal anti-inflammatory drugs [↑](#footnote-ref-20)
21. Stretching Exercises [↑](#footnote-ref-21)
22. Rheumatoid Arthritis [↑](#footnote-ref-22)
23. Forkhead box P3 [↑](#footnote-ref-23)
24. Retinoic acid-related orphan receptor gamma t [↑](#footnote-ref-24)
25. T-Box Transcription Factor 21 [↑](#footnote-ref-25)
26. Osteoarthritis [↑](#footnote-ref-26)
27. Morning sickness [↑](#footnote-ref-27)
28. Glycemic indices [↑](#footnote-ref-28)
29. C-Reactive Protein [↑](#footnote-ref-29)
30. Endothelial dysfunction [↑](#footnote-ref-30)
31. Open-label randomized clinical trial [↑](#footnote-ref-31)
32. Polymerase Chain Reaction [↑](#footnote-ref-32)
33. Chest Computed tomography scan [↑](#footnote-ref-33)
34. Complete blood count [↑](#footnote-ref-34)
35. C-reactive protein [↑](#footnote-ref-35)
36. Lactate dehydrogenase [↑](#footnote-ref-36)
37. Erythrocyte sedimentation rate [↑](#footnote-ref-37)
